# A triclinic polymorph of 4-cyano­pyridinium hydrogen chloranilate

**DOI:** 10.1107/S1600536812037221

**Published:** 2012-09-01

**Authors:** Kazuma Gotoh, Hiroyuki Ishida

**Affiliations:** a Department of Chemistry, Faculty of Science, Okayama University, Okayama 700-8530, Japan

## Abstract

The asymmetric unit of the triclinic polymorph of the title compound (systematic name: 4-cyano­pyridinium 2,5-dichloro-4-hy­droxy-3,6-dioxocyclo­hexa-1,4-dien-1-olate), C_6_H_5_N_2_^+^·C_6_HCl_2_O_4_^−^, consists of two crystallographically independent cation–anion units, in each of which the cation and the anion are linked by an N—H⋯O hydrogen bond. In the units, the dihedral angles between the cation and anion rings are 78.43 (11) and 80.71 (11)°. In the crystal, each unit independently forms a chain through N—H⋯O and O—H⋯N hydrogen bonds; one chain runs along the *c* axis while the other runs along [011]. Weak C—H⋯O, C—H⋯N and C—H⋯Cl inter­actions are observed between the chains.

## Related literature

For the monoclinic polymorph, see: Tomura & Yamashita (2008 ▶); Gotoh *et al.* (2008 ▶). For hydrogen-bonding patterns in chloranilic acid–organic base (1/1) systems, see: Ishida & Kashino (2002 ▶). For ^35^Cl nuclear quadrupole resonance studies on proton transfer in chloranilic acid–organic base systems, see: Nihei *et al.* (2000 ▶).
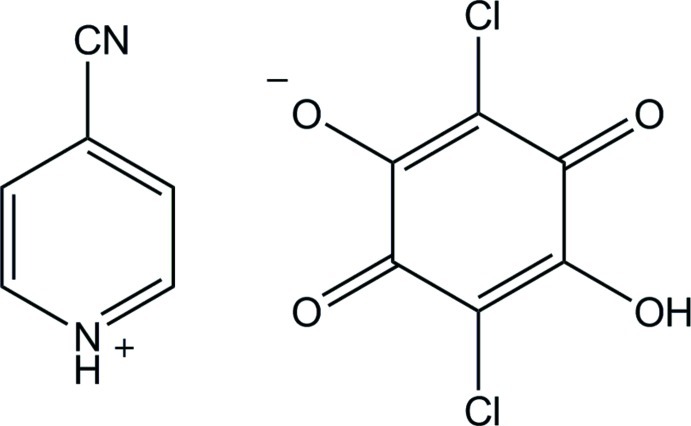


## Experimental

### 

#### Crystal data

C_6_H_5_N_2_^+^·C_6_HCl_2_O_4_^−^
*M* *_r_* = 313.10
Triclinic, 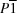

*a* = 9.3918 (7) Å
*b* = 10.6652 (7) Å
*c* = 13.9135 (8) Å
α = 111.8033 (18)°
β = 106.258 (3)°
γ = 90.416 (3)°
*V* = 1232.50 (14) Å^3^
*Z* = 4
Mo *K*α radiation
μ = 0.54 mm^−1^
*T* = 180 K
0.36 × 0.31 × 0.09 mm

#### Data collection

Rigaku R-AXIS RAPID II diffractometer
Absorption correction: numerical (*NUMABS*; Higashi, 1999 ▶) *T*_min_ = 0.866, *T*_max_ = 0.953
21354 measured reflections
7135 independent reflections
4934 reflections with *I* > 2σ(*I*)
*R*_int_ = 0.108

#### Refinement

*R*[*F* ^2^ > 2σ(*F* ^2^)] = 0.066
*wR*(*F* ^2^) = 0.164
*S* = 0.99
7135 reflections
377 parameters
H atoms treated by a mixture of independent and constrained refinement
Δρ_max_ = 0.89 e Å^−3^
Δρ_min_ = −0.83 e Å^−3^

### 

Data collection: *PROCESS-AUTO* (Rigaku/MSC, 2004 ▶); cell refinement: *PROCESS-AUTO*; data reduction: *CrystalStructure* (Rigaku/MSC, 2004 ▶); program(s) used to solve structure: *SHELXS97* (Sheldrick, 2008 ▶); program(s) used to refine structure: *SHELXL97* (Sheldrick, 2008 ▶); molecular graphics: *ORTEP-3* (Farrugia, 1997 ▶); software used to prepare material for publication: *SHELXL97* and *PLATON* (Spek, 2009 ▶).

## Supplementary Material

Crystal structure: contains datablock(s) global, I. DOI: 10.1107/S1600536812037221/lh5521sup1.cif

Structure factors: contains datablock(s) I. DOI: 10.1107/S1600536812037221/lh5521Isup2.hkl

Supplementary material file. DOI: 10.1107/S1600536812037221/lh5521Isup3.cml

Additional supplementary materials:  crystallographic information; 3D view; checkCIF report

## Figures and Tables

**Table 1:** Hydrogen-bond geometry (Å, °)

*D*—H⋯*A*	*D*—H	H⋯*A*	*D*⋯*A*	*D*—H⋯*A*
N1—H1⋯O2	0.95 (4)	1.67 (4)	2.602 (2)	170 (3)
N3—H3⋯O6	0.90 (4)	1.84 (4)	2.719 (3)	166 (3)
O4—H4⋯N2^i^	0.80 (4)	1.99 (4)	2.741 (3)	155 (4)
O8—H8⋯N4^ii^	0.93 (5)	2.07 (5)	2.875 (3)	144 (4)
C13—H13⋯N4^iii^	0.95	2.55	3.407 (3)	150
C14—H14⋯O3^iv^	0.95	2.42	3.209 (3)	141
C16—H16⋯Cl3^v^	0.95	2.71	3.431 (3)	133
C17—H17⋯O1^vi^	0.95	2.30	3.194 (3)	157
C19—H19⋯O2	0.95	2.25	3.192 (3)	170
C20—H20⋯Cl1	0.95	2.83	3.626 (3)	142
C23—H23⋯O5^vii^	0.95	2.14	3.040 (3)	158

Symmetry codes: (i) 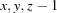; (ii) 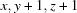; (iii) 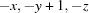; (iv) 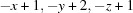; (v) 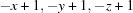; (vi) 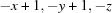; (vii) 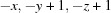.

**Fig. 1.**
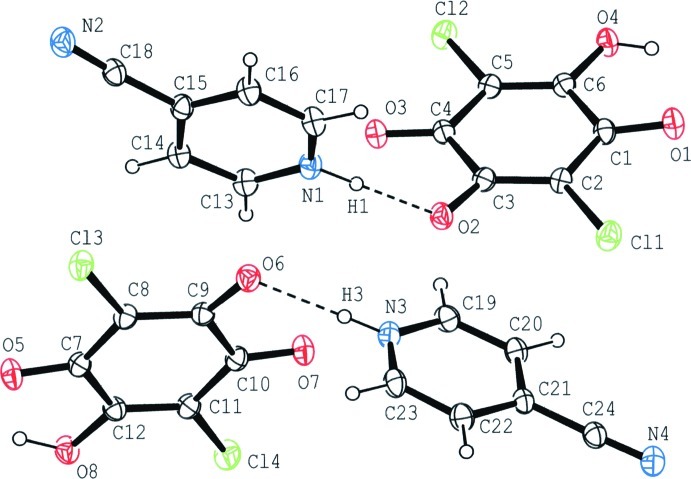
The asymmetric unit of the title compound, with the atom-labeling. Displacement ellipsoids of non-H atoms are drawn at the 50% probability level. The dashed lines indicate the N—H···O hydrogen bonds.

**Fig. 2.**
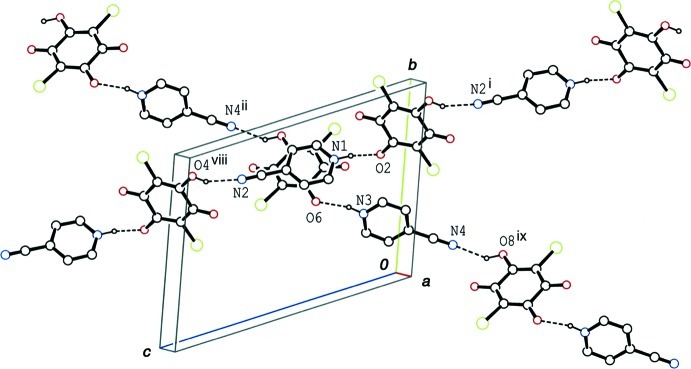
A packing diagram of the title compound, showing two crystallographically independent chains formed by N—H···O and O—H···N hydrogen bonds (dashed lines). H atoms not involved in the hydrogen bonds have been omitted. [Symmetry codes: (i) x, y, z - 1; (ii) x, y + 1, z + 1; (viii) x, y, z + 1; (ix) x, y - 1, z - 1.]

## supplementary crystallographic information

### Comment

The title compound was accidentally obtained in the preparation of the monoclinic polymorph of 4-cyanopyridinium chloranilate, C_6_H_5_N_2_^+^.C_6_HCl_2_O_4_^-^, which is an interesting model compound for investigating proton transfer in the hydrogen bond systems (Nihei *et al.*, 2000). The structure of the monoclinic polymorph has been reported by Tomura & Yamashita (2008) and Gotoh *et al.* (2008).

The asymmetric unit of the triclinic polymorph of the title compound consists of two crystallographically independent cation–anion units, in each of which the cation and the anion are held together by N—H···O hydrogen bond. The dihedral angle between the N1/C13–C17 pyridine ring and the C1–C6 of the acid ring is 78.43 (11)° in one unit, while the angle between the N3/C19–C23 and C7–C12 rings is 80.71 (11)° in the other unit.

In the crystal structure of the monoclinic polymorph, the acid molecule (A) and the base molecule (B) afford an centrosymmetric 2:2 (B:A:A:B) aggregate (Ishida & Kashino, 2002) through O—H···O and N···H···O hydrogen bonds and the H atom in the N···H···O hydrogen bond is disordered over two positions (Gotoh *et al.*, 2008). In contrast to the monoclinic form, the present triclinic polymorph shows two crystallographically independent 1:1 (A:B) units of the acid and base molecules. Each unit independently forms a hydrogen-bonded (–A:B:A:B–) chain; one chain formed by N1—H1···O2 and O4—H4···N2^i^ (symmetry code in Table 1) hydrogen bonds runs along the *c* axis, while the other chain formed by N3—H3···O6 and O8—H8···N4^ii^ (symmetry code in Table 1) hydrogen bonds runs along the [011] direction. No H atom disorder is observed in these hydrogen bonds. Between the chains, C—H···O, C—H···N and C—H···Cl interactions (Table 1) are observed.

### Experimental

Single crystals were obtained by slow evaporation from an acetonitrile solution (130 ml) of chloranilic acid (0.60 g) and 4-cyanopyridine (0.30 g) at room temperature.

### Refinement

C-bound H atoms were positioned geometrically (C—H = 0.95 Å) and refined as riding, with *U*_iso_(H) = 1.2*U*_eq_(C). The O– and N-bound H atoms were found in a difference Fourier map and refined freely. The refined distances are O—H = 0.80 (4) and 0.93 (5) Å, and N—H = 0.90 (4) and 0.95 (4) Å.

### Crystal data

**Table:**

C_6_H_5_N_2_^+^·C_6_HCl_2_O_4_^−^	*Z* = 4
*M**_r_* = 313.10	*F*(000) = 632.00
Triclinic, *P*1	*D*_x_ = 1.687 Mg m^−^^3^
Hall symbol: -P 1	Mo *K*α radiation, λ = 0.71075 Å
*a* = 9.3918 (7) Å	Cell parameters from 17217 reflections
*b* = 10.6652 (7) Å	θ = 3.1–30.1°
*c* = 13.9135 (8) Å	µ = 0.54 mm^−^^1^
α = 111.8033 (18)°	*T* = 180 K
β = 106.258 (3)°	Platelet, brown
γ = 90.416 (3)°	0.36 × 0.31 × 0.09 mm
*V* = 1232.50 (14) Å^3^	

### Data collection

**Table:**

Rigaku R-AXIS RAPID II diffractometer	4934 reflections with *I* > 2σ(*I*)
ω scans	*R*_int_ = 0.108
Absorption correction: numerical (*NUMABS*; Higashi, 1999)	θ_max_ = 30.0°
*T*_min_ = 0.866, *T*_max_ = 0.953	*h* = −13→13
21354 measured reflections	*k* = −14→14
7135 independent reflections	*l* = −19→19

### Refinement

**Table:**

Refinement on *F*^2^	Primary atom site location: structure-invariant direct methods
Least-squares matrix: full	Secondary atom site location: difference Fourier map
*R*[*F*^2^ > 2σ(*F*^2^)] = 0.066	Hydrogen site location: inferred from neighbouring sites
*wR*(*F*^2^) = 0.164	H atoms treated by a mixture of independent and constrained refinement
*S* = 0.99	*w* = 1/[σ^2^(*F*_o_^2^) + (0.0762*P*)^2^] where *P* = (*F*_o_^2^ + 2*F*_c_^2^)/3
7135 reflections	(Δ/σ)_max_ = 0.001
377 parameters	Δρ_max_ = 0.89 e Å^−^^3^
0 restraints	Δρ_min_ = −0.83 e Å^−^^3^

### Special details

**Table:**

Geometry. All e.s.d.'s (except the e.s.d. in the dihedral angle between two l.s. planes) are estimated using the full covariance matrix. The cell e.s.d.'s are taken into account individually in the estimation of e.s.d.'s in distances, angles and torsion angles; correlations between e.s.d.'s in cell parameters are only used when they are defined by crystal symmetry. An approximate (isotropic) treatment of cell e.s.d.'s is used for estimating e.s.d.'s involving l.s. planes.
Refinement. Refinement of *F*^2^ against ALL reflections. The weighted *R*-factor *wR* and goodness of fit *S* are based on *F*^2^, conventional *R*-factors *R* are based on *F*, with *F* set to zero for negative *F*^2^. The threshold expression of *F*^2^ > σ(*F*^2^) is used only for calculating *R*-factors(gt) *etc*. and is not relevant to the choice of reflections for refinement. *R*-factors based on *F*^2^ are statistically about twice as large as those based on *F*, and *R*- factors based on ALL data will be even larger.

### Fractional atomic coordinates and isotropic or equivalent isotropic displacement parameters (Å^2^)

**Table:**

	*x*	*y*	*z*	*U*_iso_*/*U*_eq_	
Cl1	0.29193 (7)	0.49544 (6)	−0.10659 (5)	0.04057 (16)	
Cl2	0.68163 (7)	1.06889 (5)	0.18401 (5)	0.03666 (15)	
Cl3	0.21149 (7)	0.59716 (6)	0.62343 (5)	0.03851 (16)	
Cl4	−0.16421 (7)	0.85552 (7)	0.30375 (5)	0.04279 (17)	
O1	0.51735 (19)	0.64141 (17)	−0.15370 (13)	0.0396 (4)	
O2	0.32328 (17)	0.66708 (16)	0.12870 (12)	0.0346 (3)	
O3	0.48242 (18)	0.91360 (17)	0.24401 (13)	0.0363 (4)	
O4	0.67459 (19)	0.88479 (17)	−0.03878 (14)	0.0343 (4)	
O5	−0.01092 (19)	0.79448 (17)	0.65480 (13)	0.0384 (4)	
O6	0.19311 (18)	0.52007 (16)	0.38448 (13)	0.0364 (4)	
O7	0.04975 (18)	0.65162 (17)	0.26162 (13)	0.0370 (4)	
O8	−0.16121 (19)	0.90831 (17)	0.53085 (14)	0.0376 (4)	
N1	0.4187 (2)	0.7224 (2)	0.33632 (16)	0.0344 (4)	
N2	0.6554 (2)	0.7841 (2)	0.74522 (17)	0.0424 (5)	
N3	0.0861 (2)	0.3707 (2)	0.16703 (16)	0.0341 (4)	
N4	−0.1526 (2)	0.0443 (2)	−0.24595 (17)	0.0413 (5)	
C1	0.4981 (2)	0.7009 (2)	−0.06484 (17)	0.0298 (4)	
C2	0.3989 (2)	0.6506 (2)	−0.02287 (17)	0.0302 (4)	
C3	0.3974 (2)	0.7129 (2)	0.08301 (17)	0.0296 (4)	
C4	0.4895 (2)	0.8540 (2)	0.15269 (17)	0.0289 (4)	
C5	0.5819 (2)	0.9100 (2)	0.10598 (17)	0.0296 (4)	
C6	0.5880 (2)	0.8389 (2)	0.00544 (17)	0.0292 (4)	
C7	0.0119 (2)	0.7548 (2)	0.56596 (17)	0.0301 (4)	
C8	0.1100 (2)	0.6605 (2)	0.53026 (18)	0.0306 (4)	
C9	0.1187 (2)	0.6129 (2)	0.42538 (17)	0.0292 (4)	
C10	0.0303 (2)	0.6811 (2)	0.35009 (17)	0.0305 (4)	
C11	−0.0679 (2)	0.7803 (2)	0.38836 (18)	0.0319 (4)	
C12	−0.0762 (2)	0.8166 (2)	0.48929 (18)	0.0301 (4)	
C13	0.3814 (3)	0.8248 (2)	0.41276 (19)	0.0354 (5)	
H13	0.3173	0.8851	0.3923	0.042*	
C14	0.4348 (3)	0.8434 (2)	0.52031 (19)	0.0338 (5)	
H14	0.4081	0.9150	0.5750	0.041*	
C15	0.5302 (3)	0.7526 (2)	0.54595 (18)	0.0321 (4)	
C16	0.5662 (3)	0.6461 (2)	0.46590 (19)	0.0378 (5)	
H16	0.6298	0.5841	0.4839	0.045*	
C17	0.5075 (3)	0.6327 (2)	0.3596 (2)	0.0381 (5)	
H17	0.5296	0.5603	0.3030	0.046*	
C18	0.5972 (3)	0.7707 (2)	0.65759 (19)	0.0357 (5)	
C19	0.1208 (3)	0.4009 (2)	0.09031 (19)	0.0359 (5)	
H19	0.1878	0.4798	0.1103	0.043*	
C20	0.0589 (3)	0.3172 (2)	−0.01748 (18)	0.0342 (5)	
H20	0.0824	0.3372	−0.0728	0.041*	
C21	−0.0390 (2)	0.2024 (2)	−0.04369 (17)	0.0303 (4)	
C22	−0.0737 (3)	0.1742 (2)	0.03736 (19)	0.0355 (5)	
H22	−0.1409	0.0965	0.0200	0.043*	
C23	−0.0083 (3)	0.2619 (2)	0.14348 (19)	0.0363 (5)	
H23	−0.0304	0.2448	0.2004	0.044*	
C24	−0.1034 (3)	0.1137 (2)	−0.15641 (18)	0.0340 (5)	
H1	0.384 (3)	0.713 (3)	0.263 (3)	0.063 (9)*	
H3	0.121 (3)	0.432 (3)	0.236 (3)	0.056 (9)*	
H4	0.665 (4)	0.833 (4)	−0.100 (3)	0.068 (11)*	
H8	−0.140 (4)	0.918 (4)	0.603 (4)	0.097 (13)*	

### Atomic displacement parameters (Å^2^)

**Table:**

	*U*^11^	*U*^22^	*U*^33^	*U*^12^	*U*^13^	*U*^23^
Cl1	0.0498 (4)	0.0352 (3)	0.0327 (3)	−0.0051 (3)	0.0117 (3)	0.0097 (2)
Cl2	0.0453 (3)	0.0310 (3)	0.0314 (3)	−0.0010 (2)	0.0092 (2)	0.0116 (2)
Cl3	0.0490 (3)	0.0387 (3)	0.0311 (3)	0.0160 (3)	0.0107 (3)	0.0180 (2)
Cl4	0.0500 (4)	0.0509 (4)	0.0361 (3)	0.0186 (3)	0.0126 (3)	0.0265 (3)
O1	0.0532 (10)	0.0367 (9)	0.0316 (8)	0.0031 (8)	0.0210 (8)	0.0102 (7)
O2	0.0398 (9)	0.0382 (8)	0.0290 (8)	0.0011 (7)	0.0142 (7)	0.0139 (7)
O3	0.0433 (9)	0.0379 (8)	0.0292 (8)	0.0064 (7)	0.0169 (7)	0.0104 (7)
O4	0.0431 (9)	0.0355 (8)	0.0282 (8)	0.0033 (7)	0.0172 (7)	0.0119 (7)
O5	0.0533 (10)	0.0417 (9)	0.0305 (8)	0.0177 (8)	0.0226 (8)	0.0181 (7)
O6	0.0396 (9)	0.0368 (8)	0.0322 (8)	0.0112 (7)	0.0112 (7)	0.0123 (7)
O7	0.0490 (10)	0.0388 (9)	0.0289 (8)	0.0111 (7)	0.0183 (7)	0.0145 (7)
O8	0.0428 (9)	0.0399 (9)	0.0348 (9)	0.0173 (7)	0.0156 (8)	0.0168 (8)
N1	0.0418 (11)	0.0349 (9)	0.0277 (9)	0.0021 (8)	0.0118 (8)	0.0127 (8)
N2	0.0492 (12)	0.0501 (12)	0.0346 (10)	0.0079 (10)	0.0168 (10)	0.0210 (10)
N3	0.0393 (10)	0.0356 (10)	0.0286 (9)	0.0110 (8)	0.0114 (9)	0.0127 (9)
N4	0.0495 (12)	0.0425 (11)	0.0331 (10)	0.0096 (9)	0.0152 (10)	0.0139 (9)
C1	0.0364 (11)	0.0292 (10)	0.0274 (10)	0.0085 (9)	0.0120 (9)	0.0132 (9)
C2	0.0349 (11)	0.0279 (10)	0.0267 (10)	0.0035 (9)	0.0089 (9)	0.0099 (9)
C3	0.0318 (11)	0.0303 (10)	0.0281 (10)	0.0046 (8)	0.0080 (9)	0.0135 (9)
C4	0.0317 (10)	0.0310 (10)	0.0277 (10)	0.0094 (9)	0.0111 (9)	0.0138 (9)
C5	0.0326 (11)	0.0284 (10)	0.0286 (10)	0.0042 (8)	0.0088 (9)	0.0123 (9)
C6	0.0316 (10)	0.0303 (10)	0.0288 (10)	0.0059 (8)	0.0093 (9)	0.0148 (9)
C7	0.0332 (11)	0.0297 (10)	0.0286 (10)	0.0038 (9)	0.0080 (9)	0.0138 (9)
C8	0.0345 (11)	0.0304 (10)	0.0289 (10)	0.0070 (9)	0.0073 (9)	0.0153 (9)
C9	0.0299 (10)	0.0291 (10)	0.0288 (10)	0.0049 (8)	0.0084 (9)	0.0118 (9)
C10	0.0331 (11)	0.0312 (10)	0.0278 (10)	0.0026 (9)	0.0081 (9)	0.0129 (9)
C11	0.0346 (11)	0.0355 (11)	0.0291 (10)	0.0064 (9)	0.0078 (9)	0.0177 (9)
C12	0.0315 (11)	0.0293 (10)	0.0315 (11)	0.0068 (9)	0.0113 (9)	0.0127 (9)
C13	0.0405 (12)	0.0333 (11)	0.0360 (12)	0.0060 (10)	0.0126 (10)	0.0166 (10)
C14	0.0408 (12)	0.0313 (10)	0.0313 (10)	0.0069 (9)	0.0149 (10)	0.0114 (9)
C15	0.0367 (11)	0.0345 (11)	0.0284 (10)	0.0023 (9)	0.0116 (9)	0.0147 (9)
C16	0.0463 (13)	0.0356 (11)	0.0362 (12)	0.0108 (10)	0.0175 (11)	0.0155 (10)
C17	0.0472 (13)	0.0363 (12)	0.0346 (11)	0.0093 (10)	0.0193 (11)	0.0128 (10)
C18	0.0407 (12)	0.0390 (12)	0.0338 (11)	0.0078 (10)	0.0166 (10)	0.0173 (10)
C19	0.0383 (12)	0.0352 (11)	0.0371 (12)	0.0039 (9)	0.0138 (10)	0.0158 (10)
C20	0.0409 (12)	0.0360 (11)	0.0313 (11)	0.0061 (10)	0.0159 (10)	0.0156 (10)
C21	0.0354 (11)	0.0322 (10)	0.0276 (10)	0.0093 (9)	0.0133 (9)	0.0136 (9)
C22	0.0435 (13)	0.0348 (11)	0.0346 (11)	0.0047 (10)	0.0162 (10)	0.0172 (10)
C23	0.0465 (13)	0.0400 (12)	0.0311 (11)	0.0113 (10)	0.0170 (10)	0.0194 (10)
C24	0.0373 (12)	0.0378 (11)	0.0317 (11)	0.0083 (10)	0.0140 (10)	0.0160 (10)

### Geometric parameters (Å, º)

**Table:**

Cl1—C2	1.730 (2)	C4—C5	1.460 (3)
Cl2—C5	1.722 (2)	C5—C6	1.339 (3)
Cl3—C8	1.736 (2)	C7—C8	1.416 (3)
Cl4—C11	1.721 (2)	C7—C12	1.514 (3)
O1—C1	1.228 (3)	C8—C9	1.382 (3)
O2—C3	1.265 (2)	C9—C10	1.551 (3)
O3—C4	1.213 (2)	C10—C11	1.457 (3)
O4—C6	1.333 (2)	C11—C12	1.336 (3)
O4—H4	0.80 (4)	C13—C14	1.374 (3)
O5—C7	1.231 (2)	C13—H13	0.9500
O6—C9	1.267 (3)	C14—C15	1.399 (3)
O7—C10	1.219 (2)	C14—H14	0.9500
O8—C12	1.334 (3)	C15—C16	1.386 (3)
O8—H8	0.93 (4)	C15—C18	1.440 (3)
N1—C13	1.341 (3)	C16—C17	1.377 (3)
N1—C17	1.344 (3)	C16—H16	0.9500
N1—H1	0.95 (3)	C17—H17	0.9500
N2—C18	1.141 (3)	C19—C20	1.376 (3)
N3—C23	1.334 (3)	C19—H19	0.9500
N3—C19	1.341 (3)	C20—C21	1.393 (3)
N3—H3	0.90 (3)	C20—H20	0.9500
N4—C24	1.141 (3)	C21—C22	1.387 (3)
C1—C2	1.424 (3)	C21—C24	1.442 (3)
C1—C6	1.512 (3)	C22—C23	1.375 (3)
C2—C3	1.377 (3)	C22—H22	0.9500
C3—C4	1.543 (3)	C23—H23	0.9500
			
C6—O4—H4	111 (2)	C12—C11—C10	120.14 (19)
C12—O8—H8	105 (2)	C12—C11—Cl4	121.12 (18)
C13—N1—C17	122.6 (2)	C10—C11—Cl4	118.65 (16)
C13—N1—H1	120 (2)	O8—C12—C11	123.68 (19)
C17—N1—H1	118 (2)	O8—C12—C7	114.58 (18)
C23—N3—C19	122.6 (2)	C11—C12—C7	121.7 (2)
C23—N3—H3	119.4 (19)	N1—C13—C14	120.7 (2)
C19—N3—H3	118 (2)	N1—C13—H13	119.6
O1—C1—C2	125.5 (2)	C14—C13—H13	119.6
O1—C1—C6	116.86 (18)	C13—C14—C15	117.2 (2)
C2—C1—C6	117.66 (19)	C13—C14—H14	121.4
C3—C2—C1	122.6 (2)	C15—C14—H14	121.4
C3—C2—Cl1	120.10 (16)	C16—C15—C14	121.4 (2)
C1—C2—Cl1	116.92 (17)	C16—C15—C18	118.3 (2)
O2—C3—C2	126.0 (2)	C14—C15—C18	120.2 (2)
O2—C3—C4	115.97 (19)	C17—C16—C15	118.3 (2)
C2—C3—C4	117.96 (18)	C17—C16—H16	120.9
O3—C4—C5	122.7 (2)	C15—C16—H16	120.9
O3—C4—C3	118.70 (18)	N1—C17—C16	119.8 (2)
C5—C4—C3	118.59 (18)	N1—C17—H17	120.1
C6—C5—C4	120.3 (2)	C16—C17—H17	120.1
C6—C5—Cl2	121.68 (16)	N2—C18—C15	177.4 (3)
C4—C5—Cl2	117.99 (16)	N3—C19—C20	119.8 (2)
O4—C6—C5	122.1 (2)	N3—C19—H19	120.1
O4—C6—C1	115.73 (19)	C20—C19—H19	120.1
C5—C6—C1	122.17 (18)	C19—C20—C21	118.5 (2)
O5—C7—C8	127.07 (19)	C19—C20—H20	120.7
O5—C7—C12	114.44 (19)	C21—C20—H20	120.7
C8—C7—C12	118.49 (19)	C22—C21—C20	120.5 (2)
C9—C8—C7	122.90 (18)	C22—C21—C24	120.7 (2)
C9—C8—Cl3	120.81 (16)	C20—C21—C24	118.83 (19)
C7—C8—Cl3	116.14 (16)	C23—C22—C21	118.2 (2)
O6—C9—C8	126.72 (19)	C23—C22—H22	120.9
O6—C9—C10	116.38 (18)	C21—C22—H22	120.9
C8—C9—C10	116.90 (18)	N3—C23—C22	120.4 (2)
O7—C10—C11	122.69 (19)	N3—C23—H23	119.8
O7—C10—C9	117.86 (19)	C22—C23—H23	119.8
C11—C10—C9	119.41 (18)	N4—C24—C21	178.9 (3)
			
O1—C1—C2—C3	170.6 (2)	O6—C9—C10—O7	−7.3 (3)
C6—C1—C2—C3	−9.2 (3)	C8—C9—C10—O7	171.9 (2)
O1—C1—C2—Cl1	−2.5 (3)	O6—C9—C10—C11	174.71 (19)
C6—C1—C2—Cl1	177.63 (15)	C8—C9—C10—C11	−6.1 (3)
C1—C2—C3—O2	−172.6 (2)	O7—C10—C11—C12	−174.8 (2)
Cl1—C2—C3—O2	0.3 (3)	C9—C10—C11—C12	3.1 (3)
C1—C2—C3—C4	9.6 (3)	O7—C10—C11—Cl4	1.7 (3)
Cl1—C2—C3—C4	−177.44 (15)	C9—C10—C11—Cl4	179.63 (15)
O2—C3—C4—O3	−2.2 (3)	C10—C11—C12—O8	178.7 (2)
C2—C3—C4—O3	175.76 (19)	Cl4—C11—C12—O8	2.3 (3)
O2—C3—C4—C5	177.69 (18)	C10—C11—C12—C7	−1.4 (3)
C2—C3—C4—C5	−4.4 (3)	Cl4—C11—C12—C7	−177.88 (16)
O3—C4—C5—C6	178.8 (2)	O5—C7—C12—O8	2.3 (3)
C3—C4—C5—C6	−1.1 (3)	C8—C7—C12—O8	−177.45 (19)
O3—C4—C5—Cl2	−1.2 (3)	O5—C7—C12—C11	−177.6 (2)
C3—C4—C5—Cl2	178.92 (14)	C8—C7—C12—C11	2.7 (3)
C4—C5—C6—O4	−177.88 (19)	C17—N1—C13—C14	0.8 (3)
Cl2—C5—C6—O4	2.1 (3)	N1—C13—C14—C15	0.7 (3)
C4—C5—C6—C1	1.6 (3)	C13—C14—C15—C16	−1.6 (3)
Cl2—C5—C6—C1	−178.48 (15)	C13—C14—C15—C18	176.4 (2)
O1—C1—C6—O4	2.9 (3)	C14—C15—C16—C17	1.0 (3)
C2—C1—C6—O4	−177.21 (18)	C18—C15—C16—C17	−177.1 (2)
O1—C1—C6—C5	−176.6 (2)	C13—N1—C17—C16	−1.4 (3)
C2—C1—C6—C5	3.3 (3)	C15—C16—C17—N1	0.5 (3)
O5—C7—C8—C9	174.2 (2)	C23—N3—C19—C20	−0.5 (3)
C12—C7—C8—C9	−6.1 (3)	N3—C19—C20—C21	0.0 (3)
O5—C7—C8—Cl3	−1.5 (3)	C19—C20—C21—C22	0.5 (3)
C12—C7—C8—Cl3	178.26 (15)	C19—C20—C21—C24	−179.5 (2)
C7—C8—C9—O6	−173.2 (2)	C20—C21—C22—C23	−0.4 (3)
Cl3—C8—C9—O6	2.2 (3)	C24—C21—C22—C23	179.6 (2)
C7—C8—C9—C10	7.6 (3)	C19—N3—C23—C22	0.6 (3)
Cl3—C8—C9—C10	−176.91 (15)	C21—C22—C23—N3	−0.1 (3)

### Hydrogen-bond geometry (Å, º)

**Table:**

*D*—H···*A*	*D*—H	H···*A*	*D*···*A*	*D*—H···*A*
N1—H1···O2	0.95 (4)	1.67 (4)	2.602 (2)	170 (3)
N3—H3···O6	0.90 (4)	1.84 (4)	2.719 (3)	166 (3)
O4—H4···N2^i^	0.80 (4)	1.99 (4)	2.741 (3)	155 (4)
O8—H8···N4^ii^	0.93 (5)	2.07 (5)	2.875 (3)	144 (4)
C13—H13···N4^iii^	0.95	2.55	3.407 (3)	150
C14—H14···O3^iv^	0.95	2.42	3.209 (3)	141
C16—H16···Cl3^v^	0.95	2.71	3.431 (3)	133
C17—H17···O1^vi^	0.95	2.30	3.194 (3)	157
C19—H19···O2	0.95	2.25	3.192 (3)	170
C20—H20···Cl1	0.95	2.83	3.626 (3)	142
C23—H23···O5^vii^	0.95	2.14	3.040 (3)	158

Symmetry codes: (i) *x*, *y*, *z*−1; (ii) *x*, *y*+1, *z*+1; (iii) −*x*, −*y*+1, −*z*; (iv) −*x*+1, −*y*+2, −*z*+1; (v) −*x*+1, −*y*+1, −*z*+1; (vi) −*x*+1, −*y*+1, −*z*; (vii) −*x*, −*y*+1, −*z*+1.
